# Immune Reconstitution Disorders: Spotlight on Interferons

**DOI:** 10.31531/2581-4745.1000119

**Published:** 2019

**Authors:** Hesham Mohei, Usha Kellampalli, Irina Vlasova-St. Louis

**Affiliations:** Department of Medicine, University of Minnesota, Minneapolis, USA

**Keywords:** Immune reconstitution, Type I/II interferons, allo-HSCT, IFNG, T cells, AIDS, HIV, IRIS

## Abstract

Three decades of research in hematopoietic stem cell transplantation and HIV/AIDS fields have shaped a picture of immune restoration disorders. This manuscript overviews the molecular biology of interferon networks, the molecular pathogenesis of immune reconstitution inflammatory syndrome, and post-hematopoietic stem cell transplantation immune restoration disorders (IRD). It also summarizes the effects of thymic involution on T cell diversity, and the results of the assessment of diagnostic biomarkers of IRD, and tested targeted immunomodulatory treatments

## Introduction

Alterations in the balanced recovery of innate and adaptive immunity represent the immune restoration disorders. The spectrum of them have been grown throughout the past decade, and their pathology somewhat clarified. The common theme among them is that they occurred in lymphopenic individuals, who can be divided into two large groups: 1. In patients with primary immunodeficiency state due to advanced HIV infection, who initiated antiretroviral therapies (ART), and 2. In transplant patients after high-dose chemo-radiation-therapy who underwent hematopoietic stem cell transplantation. These poorly defined conditions require prolonged infection surveillance, careful choice of anti-inflammatory medications, as well as expensive laboratory monitoring throughout the immune reconstitution phases. Because these patients experience either prolonged and severe immune deficiency due to HIV, or acute myeloablation due to treatment, the immune reconstitution kinetics differ, but the T cell immunity is particularly affected in both.

High occurrence of inflammatory immune restoration is exclusively reported in patients who initiated antiretroviral therapy after the onset of severe immunodeficiency. This disorder is reported as immune reconstitution inflammatory syndrome (IRIS), or immune reconstitution/restoration disease (IRD). IRIS is a common complication in AIDS patients who initiated ART. It can be described as a paradoxical inflammatory reaction that occurs in response to persistent opportunistic pathogens: mycobacterial, viral, fungal, or parasitic [1,2]. Among diverged clinical symptomatology, one common denominator is that the patient’s immune system begins improving due to ART and subsequently deteriorate [3]. Since the antigen-specific T cell-mediated immunity is nearly wiped out by HIV, the restoration of it after therapy initiation trigger a dysfunctional response against persisting antigens, whether present as whole microorganisms, or in the form of pathogen-associated molecules, which trigger a highly inflammatory immune activation [4].

The allogeneic hematopoietic stem cell transplantation (allo-HSCT) has proven to be lifesaving for many patients recovering from immuno-hematological malignancies [5]. Outcomes however are dependent on the eradication of malignant cells, donor compatibility, and on how successful is the reconstitution of recipients’ immuno-hematopoiesis [6]. The phases of immune reconstitution are usually assessed at several milestones, such as neutrophil engraftment, the innate immune recovery, and eventually an adaptive immune recovery, which may finalize by 24 months post-transplantation [7]. Delayed or incomplete immune reconstitution has been associated with significant morbidity and mortality, especially in adults due to infections, transplant rejection, or malignant disease relapse [8]. The restoration of T cell immune responses is increasingly recognized as the main determinant which sets apart favorable immune reconstitution from pathological one, which is accompanied by immune exhaustion, pathogenic autoimmune activation, or graft versus host reactions (GVHD) [9].

Three decades of research in transplantation and HIV fields have shaped a picture of immune restoration disorders. In the absence or poor recovery of T cells, other immune cells synergize their effort to control infections while preventing excessive inflammation or autoimmunity. Particularly, monocytes, natural killer cells (NK) or B cells are able to undergo faster homeostatic expansion and produce and overlapping repertoire of immune mediators (e.g. cytokines of interferon family). It remains unclear whether complete T cell immune reconstitution ever occurs in older individuals who are most susceptible to immune reconstitution disorders.

Thus, cumulative knowledge about innate and adaptive immune cells and the secretions of cytokines during immune recovery has an important clinical implication for assessment of favorable or the diagnosis of pathological immune reconstitution. One of the cytokine family we are going to highlight in this review is type I/II interferons, which exhibit defined kinetics during immune reconstitution phases. Type I/II interferon networks play a unique role in the connection between innate and adaptive immunity, therefore essential in normal and pathological immune reconstitution.

## Interferon Signaling

Interferons (IFNs) are a group of signaling proteins that are being made and released by immune and non-immune cells to heighten their defenses against intracellular pathogens [10]. The release of interferons causes a range of flu-like symptoms from subfebrile fever, mild muscle or body aches, to cytokine storm-like symptoms when IFNs are overproduced along with other cytokines and pro-inflammatory molecules resulting in multiple organ failure. IFNs play a dual role in inflammation: pro- and anti-inflammatory [11]. IFNs enhance antigen presentation by virtue of increasing the expression of human leukocyte antigens (HLA) on antigen-presenting cells and enhancing lysis potentials of natural killer (NK) cells [12]. Interferons display protective anti-inflammatory functions via direct inhibition of pro-inflammatory cytokines, inductions of cytokine antagonists or re-directing the signaling through negative feedback loops [13]. All types of IFNs bind to a specific heteromeric cell surface receptor complexes known as the IFN receptors (IFNR) that play a crucial role in direction of many cellular processes toward pro- or anti-inflammatory outcomes [14]. Based on the type of receptor through which they signal, human interferons have been classified into three major types [15,16].

## Interferon Type I, III

IFNs are long known to inhibit viral replication in virus-infected cells, thus represent one of the most important anti-viral innate immunity defenses [17,18]. Additionally, type I IFNs play a significant role in the response to bacterial infections [19]. Type I IFNs are composed of IFNB, IFNA that can further be classified into 13 different subtypes, and the extended family of IFN E/K/W [18]. A number of cells produce IFNA and IFNB, including macrophages/monocytes, fibroblasts, and endothelial cells also called natural interferon-producing cells. Additionally, plasmacytoid dendritic cells (pDCs) are able to secrete up to one thousand times more interferons type I than the others above [20].

Type I IFNs and more recently discovered type III IFNs (IFN-lambda, IFNL1–4) signaling rely on a group of intracellular transcription factors STAT 1–4 (signal transducer and activator of transcription 1–4). Activated through IFN receptors, these cells quickly secrete type I IFNs to enhance the cytotoxic function of NK, B, and T cells, which links innate and adaptive immune responses. Type I IFN binds to IFNAR receptor and activate a robust transcriptional pathway through a JAK-STAT signaling, interferon response factors (IRF 1–9), or partially overlapping but distinct interferon stimulated genes (ISGs), to action [21,22]. The transcriptional complexes activated by type I IFN signaling bind to specific interferon stimulating responsive element (ISRE) and gamma interferon activation sites (GAS) sequences within promoter regions of ISGs and lead to the expression of numerous genes important for cell death, cell proliferation and immune responses [23].

Although the specifics of IFN immune activation are complex, it appears to begin with activation of innate phagocytic leukocytes by antigens. Type I IFN synthesis is induced by two groups of antigenic challenges: the pathogen-associated molecular patterns (PAMPs) and danger-associated molecular pattern (DAMPs) through the pattern recognition receptors (PRRs). These antigen-sensor receptors can be found in the cytosol or in the endosome of cells. The signal is transmitted through four smaller networks of PRRs:

1. Toll-like receptors, TLRs1–11.
2. Nucleotide-binding oligomerization domain (NOD)-like receptors, NLRs.
3. Retinoic acid-inducible gene-1 (RIG1)-like receptors, RLR.
4. C-type lectin receptors, CLRs.

All these receptors utilize the common downstream serine/threonine protein kinases (e.g. TBK1 and IKKE) to transfer signals to IFN regulatory factors (IRFs) and nuclear factor kappa B (NFkB), and thus activating type I IFN transcription.

TLRs are cytoplasmic and endosomal receptors that are specialized in detecting specific PAMP molecules. For example, expression of type I and III IFNs can be induced upon recognition of viral components by TLR3, 7, or 9. TLR signaling is transmitted through TRIF (TRL3 and TLR4) or Myd88 (TLR4, 7, 8, and 9), and multiple IRFs to activate transcriptions of IFNA and IFNB in infected cell [24]. NLRs family is also activated by the same PAMPs (e.g. pathogen derived lipopolysaccharides, peptidoglycans, glycoproteins, RNA and DNA). The NOD2, for example, activates the downstream TBK1 and IRF5 signaling pathway, leading to the production of type I IFN [25].

However, NLR signaling frequently leads to the synthesis of absent in melanoma 2 (AIM2), interleukin 1 beta (IL1B) and IL18, the protein products of NLRP3-inflammasome activation. NLRP3 (NOD-like receptor family pyrin domain containing 3) pathway has been reported to negatively regulate the type I IFN production and set a pro-inflammatory state accompanied by necrosis [26]. Thus, NLR-inflammasome represents an alternative (to interferon type II) and very damaging route of responses to PAMPs [27]. The RLR activation by RNA viruses in numerous non-immune cells also leads to type I and III interferon production [28,29]. The upregulation of interferon and interferon response pathways helps limit viral replication, but since these defense pathways are accompanied by apoptosis it may contribute to slow immune reconstitution and immune complication, such as IRIS [30].

Recent information demonstrates the importance of type III IFNs in not only viral [31–33], but also in some fungal infections [34]. IFNLs are potent antiviral agents, with very mild pro-inflammatory effects, since they are not expressed in macrophages [35]. This fact may suggest important, yet unknown, role of IFNL in AIDS immune reconstitution. The production of IFNA and IFNB can be induced after recognition of fungal antigens through CLR signaling and IRF5. Sensing of some bacterial and fungal pathogens by C-type lectin receptors has also been reported to induce type I IFN production by innate immune cells, which may represent a clinically relevant path to understand the pathophysiology of immune reconstitution [36].

## Interferon Type II

Type II IFN has only one representative, IFN gamma (IFNG). IFNG, originally known as the immune interferon, plays a key role in host defense against cellular and intracellular pathogens, including fungal, viral, bacterial and parasitic [37]. This cytokine plays a major role in mammalian adaptive immune responses, as it is secreted by activated CD8+ cytotoxic T cells and CD4+ T helper cells type 1 (Th1) [38]. IFNG possesses diverse biological properties, including immunomodulatory activities on innate immune cells, such as macrophages, monocytes, NK cells, and neutrophils [39,40]. The B and NK cells are also able to produce this cytokine, yet to a lesser extent [41,42].

Macrophages, activated by IFNG, exhibit improved microbial killing ability via heighten pinocytosis and phagocytosis. IFNG can also act as a cell growth inhibitor, via autocrine activation loop, directly triggering apoptosis in target cells (infected or transformed) by activating cytotoxic CD8+ T cells producing granzymes [43]. IFNG signals are transmitted via IFNG receptors (IFNGR1,2) which dictate the strength of interferon signaling [12]. Upon engagement with IFNG, IFNG receptors activate Janus-activated kinase 1 (JAK1), JAK2, and STAT1 signaling to regulate the transcription of many IFNG-inducible genes through activation of interferon-regulatory factors IRF1 and IRF2 [44,45]. The IRFs translocate to the nucleus members where they interact with interferon-stimulated response element (ISRE) to regulate expression of numerous interferon-stimulated genes (ISG) resulting in various physiological responses ranging from cell apoptosis, cell senescence to cell proliferation [46]. Involvement of IFNG in autophagy, inflammasome formation in target cells, or antibody-mediated complement activation is just beginning to be reported [47,48].

**Figure 1:**
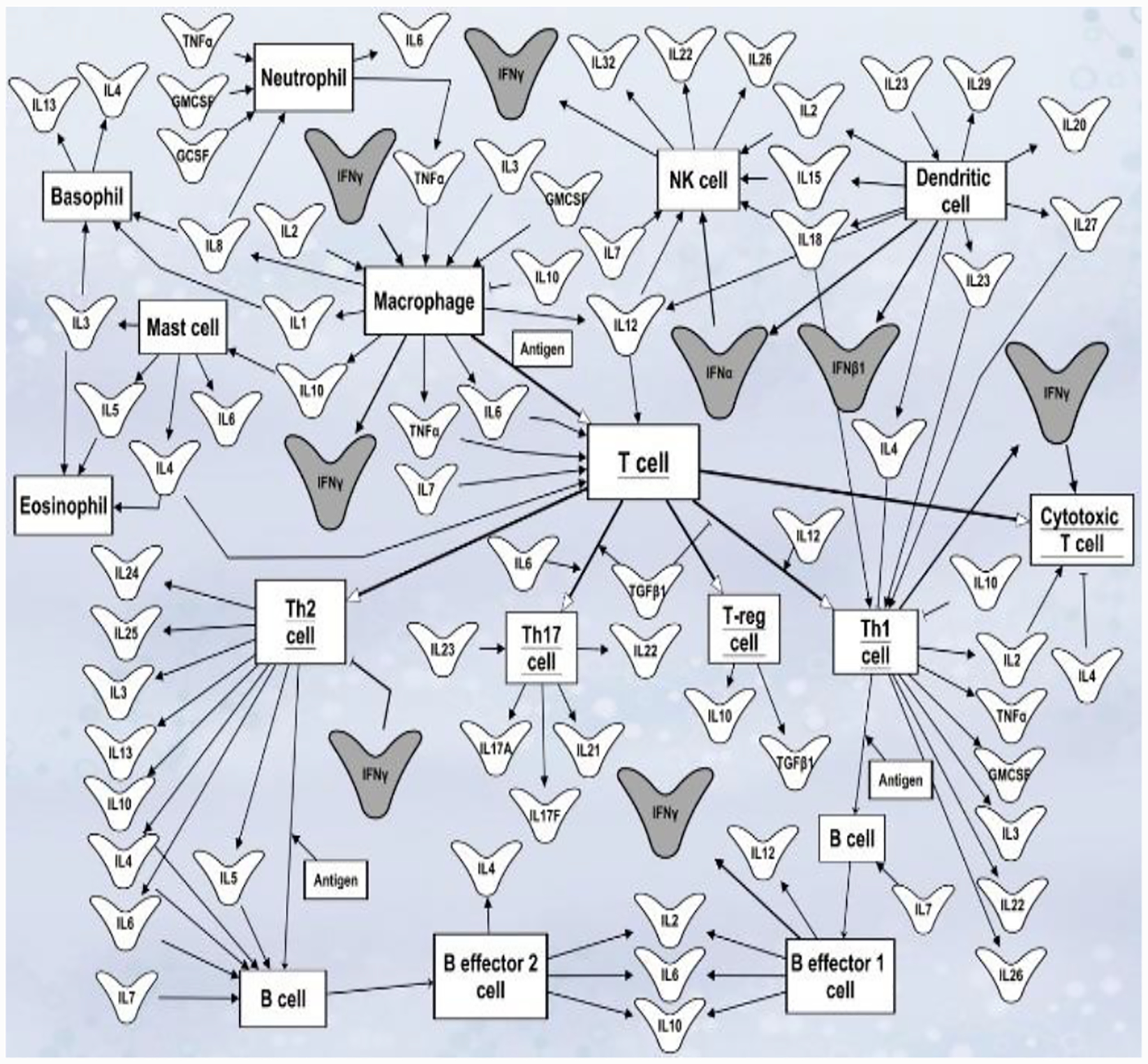
Role of cytokines in mediating communication between immune cells. Cytokines mediate cross-regulation of numerous signaling pathways between innate and adaptive immune cells. Outward arrows represent cytokine release. Point arrows represent cytokine activation function. The dull arrows represent cytokine inhibitory function. Cell’s names are shown in boxes. Interferons are highlighted in dark grey. This network diagram was built using Qiagen Pathway Assistant software.

Interferon response comprises a series of reactions that alter the expression of a variety of human genes [49]. Since interferons shared signaling molecules downstream the receptors and common transcription factors, the overall effect on target cells depends on the density of different receptors, how well intracellular signaling is transmitted, and the level of soluble IFNs produced [50,51]. Interferons are of high importance for proper communication between innate and adaptive immunity (Figure 1). IFNG, for example, induces transcription of IL15 in monocytes which in turn promote the proliferation of memory CD8+ T cells, NK, and natural killer T (NKT) cells re-direct immune responses toward pro- and anti-inflammatory depending upon cellular milieu [52]. IFNG is activated by IL12 and IL18 which are secreted by dendritic cells, monocytes, macrophages, neutrophils and epithelial cells [53].

IL27 plays an important role in naïve T cells clonal proliferation and differentiation into the Th1 lineage [54]. Cooperatively with IL12, it increases IFNG production by naïve T cells [55]. Human peripheral blood cells treated with type I IFN can increase dendritic cell maturation and IL12 production which increases priming and production of IFNG by T cells [56]. IL4 and IL10 are examples of negative regulators of IFNG production [57]. Addition of IFNB at the time of infection has been shown to negatively affect IFNG production *via* IL10-dependent- and independent mechanisms [58]. Conversely, IFNA acts synergistically with IFNG in development of T effector cells [59].

Interferons are required for communication between lymphocytes and macrophages, and play a unique role in macrophage M1-M2 polarization [60]. M1-IFNG type predominantly occurs during acute infection when proinflammatory M1 macrophages are stimulated by IFNG (along with PAMPs and TLRs). M1 type macrophages express CD86 and secrete inflammatory mediators like tumor necrosis factor alpha (TNFA), IL1B, IL6, IL8, IL12 and IL23 [61,62].

Activated M1 macrophages travel to the site of infection, induce inflammation via nitric oxide (NO) and reactive oxygen intermediates (ROI), and damage infected cells. Subsequently, polarized into M2 type by IL4, IL10, IL13, and transforming growth factor beta (TGFB), macrophages phagocytose cellular debris in order to resolve inflammation and to facilitate wound healing [63,64]. M2 macrophages abundantly express mannose receptor, dectin-1, CD163, CD209, scavenger receptor A and B1, CCR2, CXCR1, and CXCR2.

Additionally, M2 exhibit different metabolic profile: high production of ornithine and polyamines through the arginase pathway. The proper switch between M1/M2 phenotype is important to pro-resolution of inflammation, restoring Th1/Th2 balance and immune homeostasis during immune reconstitution [58,65–67]. However, inappropriate polarization drives disease pathology. In the absence of IFNG signaling (IFNR knockout), macrophage activation by type I IFNs is likely to take over, and this justify the beneficial effects of IFNA treatment in patients with compromised IFNG responses [68].

## Thymic Involution and Effect on Interferon-Producing Cells

Thymus is a vital organ of the adaptive immune system. Thymopoiesis is a fundamental route for generation and maturation of naive T cells into CD4+ helper, CD8+ cytotoxic effector and CD4+CD25+ and Forkhead Box protein 3 (FOXP3+) regulatory T cells, among others [69,70]. The bone marrow-derived T cell progenitors traverse to the thymus, become committed to the T cell lineage, and undergo proliferative expansion and maturation [69]. During thymopoiesis, the T cell receptor (TCR) diversity is generated through recombinant rearrangement of variable, diversity and joiner genes, resulting in a broad antigen-specific repertoire of T cells [71].

At every stage of T cell development and maturation, T cells are sensitive to signals from cytokines. Age-related regression of thymus is a well-known phenomenon associated with a decline in naive T cell output and changes cytokine profiles [72]. Regression escalates during chronic HIV infection, or by treatments with chemo- or radio-therapeutic agents. When peripheral T cell populations are severely depleted, a renewal of thymic activity is essential to T cell reconstitution. Thymic involution is presented as a decrease in total thymic cellularity, increase in perivascular space, and disruption of the thymic architecture, which thought to contribute to the reduction in naïve T cell diversity and restriction in the peripheral TCR repertoire [73,74]. As seen in older individuals, thymic involution is linked with increased susceptibility to infections, autoimmune diseases, and cancer. Since T lymphocytes are major producers of IFNG, the health of thymus is the utmost importance for the development of IFNG-producing cells.

The immunosuppressive nature of pre-allo-HSCT conditioning therapies is toxic to pre-thymic, thymic, and post-thymic stages of T-cell development. Following the resolution of the acute insult, the thymus is somewhat capable of intrinsic recovery in younger patients, however the restoration of thymopoiesis in adults is highly questionable [75,76]. Myeloablative conditioning (MAC) consists of high doses of radiation or chemotherapy and aims to eradicate resistant cancer cells. MAC regimens result in the destruction of bone marrow cellularity and are lethal in one hundred percent of patients without immediate hematopoietic stem cell transplantation. However, many patients are unable to tolerate MAC, even with allo-HSCT. The reduced-intensity conditioning (RIC) was offered to older patients and to those with increased comorbidities [77]. Subsequent clinical studies comparing MAC and RIC showed no significant difference in overall survival (OS) between MAC and RIC groups [78,79].

The leukemia-free survival and non-relapse mortality also did not differ significantly between the compared groups. Moreover, the cumulative incidence of chronic GVHD and all types of infectious complications were less frequent with RIC than with MAC [78,79]. Avetisyan et. al. showed that allo-HSCT recipients receiving MAC had a higher CMV viral load and weakened T cell reconstitution as detected by low IFNG production than those receiving RIC [80]. Thus, RIC regimens are more favorable toward the thymic recovery and shorten the duration of post-transplant immunodeficiency, thereby reducing susceptibility to viral infections [81].

The homeostatic expansion of the peripheral T cell pool can facilitate T cell recovery within several months post-transplantation, although clearly the TCR repertoire diversity never recovers [69,82–84]. Immunosuppressive therapies to prevent graft versus host diseases (GVHD) during the post-transplant period also impairs thymic function and thus increase the risk of infections. Anti-thymocyte globulin (ATG) regimens used to prevent GVHD after allo-HSCT showed a significantly prolonged thymic dysfunction and delayed recovery of total CD4+ T cells in ATG-treated patients [85].

The post-HSCT thymic activity has conventionally been monitored radiologically and through immunophenotyping [86,87]. Peripheral naive and memory T cells can be distinguished by their expression patterns of cell surface markers, including CD62L, CCR7, CD27, CD45RO, CD45RA, CD28, CD103, or alpha E beta 7 integrin [88]. The quantification of recent thymic emigrants (RTEs) can be detected as TCR rearrangement DNA excision circles (TRECs) [89]. TRECs are indicators of recovery of naïve and memory T cell that significantly correlate with virologic suppression and improved long-term clinical outcome in adult allo-HSCT recipients [66,90].

Thymus regression is a feature of AIDS pathogenesis. Chronic HIV infection induces a substantial suppression of thymocyte proliferation. Resulted loss of generations of naïve T cells contributes to dysbalanced immune restoration in patients commencing ART [91]. For example, the disproportionate ratio of Th17/Treg (T helper 17/FOXP3+ T regulatory) yield T cell immune responses ineffective towards opportunistic infections, enhancing pro-inflammatory state and predisposing to IRIS [92]. An abnormal overrepresentation of cells with CD127 low FOXP3+ CD25+ Tregs phenotype, due to significant expansion, and a higher ratio of Tregs to effector/memory T cells was found in IRD patients as the main contributor to dysregulated of T cell repopulation [93]. The lower absolute number of Tregs pre-ART commencement was noted in patients who later develop IRIS [94]. The defects in IL7/IL7R(CD127) pathway are maybe behind poor reconstitution of thymic cell lineages, as IL7 cytokine is essential in primary T cells development [95].

Akilimali et al. reported that dysregulated production of IL7 and expression of IL-7R lead to aberrant T cell responses to cryptococcal antigens as an underlying factor in the immunopathogenesis of IRIS [96,97]. There have been attempts to restore thymic function and improve thymopoiesis with growth hormone (GH) in HIV-infected immunodeficient adults. GH treatment is associated with the significant increase in thymic density, the number of circulating TRECs and naive CD4+ T cells within peripheral blood monocyte population (PBMC) when co-administered at the initiation of antiretroviral therapy [98]. The use of GH is limited due to arthralgia, alteration in glucose metabolism and other harmful side effects in a significant number of patients [99].

## Immune Reconstitution Inflammatory Syndrome in AIDS Patients

Normal and pathological kinetics of immune reconstitution have been observed in AIDS patients after initiation of antiretroviral therapy (ART). Unlike a normal immune system, which clears infection and returns to quiescence after being activated, the ART-related restoration of the immune function is often associated with a spike of pro-inflammatory responses to opportunistic pathogens. Immune reconstitution inflammatory syndrome (IRIS) is a prevalent complication in AIDS patients in sub- Saharan Africa and a significant cause of morbidity and mortality [100,101]. It can be described as a severe inflammatory reaction that occurs in response to numerous subclinical, latent, undiagnosed, or previously treated opportunistic infections. IRIS manifestations have multifaceted symptomatology, from meningitis or focal neurological signs to development of lymphadenopathy, pneumonitis, enlargements of Kaposi sarcoma lesions, etc. [102]. Paradoxically, IRIS symptoms develop in spite of the longitudinal decrease of HIV viral load, increased CD4+ T cell counts, and microbiologic treatment success as evidenced by improved antigen clearance [103,104]. Thus, one common denominator is that the patient’s immune system begins improving due to ART and subsequently deteriorates. The predominant morbidity occurs in patients’ populations that are co-infected with Mycobacterium tuberculosis and Cryptococcus species [105]. Therefore, AIDS clinicians and researchers are searching for better approaches to diagnose and treat these forms of IRIS.

Two common features put patients at risk for IRIS development are: 1. profound baseline immunosuppression with a median CD4+ T cell count of around 25 cells/uL; 2. high antigen burden in blood or cerebrospinal fluid (CSF) [106–108]. Few associations between levels of pro-inflammatory mediators at baseline followed by IRIS onset have been established in blood and CSF [109–111]. At presentation, IRIS is highly inflammatory, with the involvement of multiple mediators that appear systemically in blood as well as locally in organs such as lungs or CSF [110].

However, specific laboratory tests that can be used for IRIS detection have still not moved into routine patient care. The mechanisms that underlie the development of IRIS is still poorly understood. Patients with IRIS seem unable to control pathological inflammatory reactions, or properly regulate the immune activation pathways during reconstitution. Several immune pathways, including interferon regulatory pathways, are implicated in IRIS pathogenesis, suggesting a pathological switch from severe immunodepression to pronounced inflammatory state that occurs during IRIS event [112].

## Interferon Signaling in the Immunopathogenesis of AIDS and IRIS

There is a wide array of evidence for the importance of interferons in the clearance of opportunistic infections, such as cryptococcosis or tuberculosis [113–115]. In immunocompetent patients, the pathogens activate the pathogen recognition receptors, which elicit intracellular signaling cascades in immune cells that rapidly lead to the activation of transcription factors, such as nuclear factor kappa B (NFkB) and IRFs, and secretion of interferons. This ultimately shapes the adaptive immune response and coordinate the elimination of pathogens and infected cells [116]. Primary immunodeficiency state, driven by CD4+ T cell loss, results in severe impairment of all branches of the immune system. In immunocompromised patients with CD4+ T cell count <50 cells/uL, low T cell responses, as evidenced by low production of IFNG, IL8, IL6, and TNFA, leads to inefficient cytotoxic response, macrophage activation and pathogen clearance [117].

Several immune abnormalities were identified before ART commencement in patients who developed IRIS in comparison with those who did not [118]. The predominant abnormality was the down-regulation of interferon-response genes in individuals who went on to develop IRIS. Poor cellular interferon responses in CSF and paucity of anti-viral gene expression in the blood at the time of ART initiation have been previously suggested to predispose patients to IRIS [119,120] and was also associated with higher mortality from IRIS [121].

Several studies reported very low interferon levels secretion by PBMC in response to antigenic stimuli prior to and after ART initiation as predictors of subsequent IRIS events [92,122]. Low concentration of IFNG at baseline may be explained by severe depletion of interferon-producing cells. Patients who exhibit such signature may require a longer course of antimicrobial therapy before initiation of ART to achieve clearance or supplemental immunotherapy [123]. Thus, an ineffective baseline immune response characterized by low production of interferons and interferon-response genes leads to poor antigen clearance and worsen outcomes.

In advanced AIDS the innate immune defense becomes the primary branch that is able to combat opportunistic infections. However, in the absence of T cell regulation, the antigen presentation is compromised by opportunistic diseases and skewed toward alternative M2 macrophage activation pathway [124,125]. Such macrophages/monocytes are unable to secrete protective concentrations of proinflammatory chemokines and cytokines (e.g. TNFA, CCR2 or IL6) at the site of infection in an attempt to attract lymphocytes during immune restoration, thus become more permissive to infection relapse [126]. After initiation of antimicrobial therapy and ART, the reduction of microbial load may result in re-polarization of monocyte/macrophage population toward M1, however, they become hyper-reactive toward regulatory stimuli, such as IFNG [127].

## Kinetics of Immune Reconstitution on ART

The role for IFNs in AIDS immune reconstitution has been described in two studies. One was conducted in peripheral blood from advanced stage HIV-infected patients without opportunistic infections who were commenced on ART and did not exhibit IRIS. The other was conducted in the South African cohort of patients with opportunistic cryptococcal infections [118]. Both cohorts exhibited an upregulation of IFN I/II-STAT and NFkB pathways at baseline, followed by a subsequent decline as early as 4 weeks after ART initiation [128].

Similar gene expression is noticed in patients with active tuberculosis: they have initial upregulation of IFN genes that down-regulated following successful treatment [129,130]. Evidence of macrophage activation and protective levels of IL6 and IFNG, IL4, IL10, and IL17 in CSF or plasma associated with favorable baseline signature for subsequent recovery on ART [109,131]. The most plausible mechanism for this is maintaining innate host defenses in an active state through the direct stimulation of the immune system by the virus itself [132]. During favorable immune reconstitution, the longitudinal upregulation of cytokines such as IL7, IL2, HLA molecules during 12 weeks on ART reflect the effective immune recovery in lymphocyte populations and improvement of the antigen-presentation.

On the contrary, IFN pathway along with IL6 was upregulated within first few weeks on ART, and at the time of IRIS events, perhaps due to activation of cytotoxic NK cells and monocytes [103]. PBMCs from tuberculosis-IRIS patients exhibit high expression of cytotoxic mediators (perforin and granzyme B), which also suggest the involvement of cytotoxic natural killer T cells [133]. Longitudinal increase of plasma levels of IL2, IFNG, TNFA, IL17, and IL8 preceded IRIS and remained elevated at the time of IRIS [134]. It is unclear which immune cells are primarily responsible for the rise of cytokine levels. It is suggested that unbalanced restoration of T cell subpopulations and miscommunication with innate immune responses may play a role.

Majority of conducted studies described IRIS events that occurred during the first 3 weeks on ART, and thus called early IRIS. High levels of IL1, IL6, IL7, IL8, granulocyte colony stimulation factor (GCSF), or IL18 cytokines can be detected in patient’s plasma or serum at the time of early IRIS events [50,119,135]. During the first 2–3 months on ART, the gain in absolute numbers of CD4+ T cells rarely occurred more than 30 cells per microliter per month [136,137]. Thus, it is not surprising that immune activation occurs via NLR-inflammasome pathways, representing exaggerated innate cells response toward ongoing viral replication and microbial antigens.

It had been recently shown that the inflammasome pathway drives CD4+ T cell depletion in HIV-1 infection and delayed immune reconstitution [138]. Thus, inflammasome pathology seems to be behind the IRIS symptoms [139]. In IRIS patients involving the deterioration of the central nervous system, the inflammasome activation may represent a peripheral biomarker of brain inflammation that crosses the blood-brain barrier [137,140,141].

The similarity of transcriptomic biomarkers between cryptococcal IRIS and tuberculosis IRIS suggests that the symptoms of deterioration may involve activation of patrolling monocytes and neutrophils [142,143]. Triggered by PAMPs, these cells secrete pro-inflammatory mediators that can be observed in blood at the time of IRIS events [144]. Thus, during poor restoration of adaptive T cell immunity, the innate immune system activation is redirected via NLR-inflammasome route, resulting in more damage to target organs and accumulation of cellular debris.

The clinical manifestations of IRIS usually begin within the first four weeks of ART initiation; however, late presentation beyond 8 weeks has also been reported [105,145]. We recently described peripheral blood changes during cryptococcal late IRIS events that occurred after 10 weeks of immune reconstitution on ART [118]. The screening analysis revealed biomarker genes that encode a variety of molecules in T, B, NK cells and neutrophils. Significant differences in gene expression between early IRIS and late IRIS events suggest that late IRIS has distinct molecular phenotypes. High level of expression of IFNG and IL27 were discovered as biomarkers of late IRIS. In our study both Th1 and Th2 response genes were upregulated in late IRIS, other studies showed that Th2 responses were predominant [146].

The upregulation of numerous chemokines, chemokine receptors, and adhesion molecules preceded late IRIS events. The CSF chemokines expression was predictive of IRIS in other studies as well [109]. Thus, late IRIS showed a signature of heightened T cell proliferation, cytokine, and chemokine production, but delayed T cell maturation, leading to inability to resolve inflammation. The innate immune system activation is still present in late IRIS, due to impaired communication between players of activation/suppression in innate and adaptive immunity, and unresolved inflammation. The clinical implication of these findings is that the onset of late IRIS can be detected in peripheral blood through monitoring aberrant kinetics of immune reconstitution. This may provide an opportunity for intervention before clinical deterioration occurs.

## Potential Treatment Approaches for Immune Reconstitution in AIDS

During immune reconstitution in AIDS patients the chronic inflammation which has not been resolved for years, derails the protective immune responses toward damaging. The clearance of opportunistic pathogens requires robust mobilization of Th1 type immunity and sufficient production of IFNG at the site of pathology. Thus, the addition of short-course IFNG to standard treatment may be beneficial to restore impaired communication between innate and adaptive immune branches. The assessment of the efficacy of adjunctive IFNG for the treatment of HIV-associated opportunistic infections has been performed by several groups of clinical investigators.

Adjunctive IFNG (given in addition to antimicrobial treatment) has been shown to be safe, with no adverse effect on CD4+ T cell count or viral load [147,148]. This is especially important since AIDS patients showed impaired type I IFN signaling [149]. A recent pre-clinical study provided evidence for the development of macrophage innate memory through IFNG priming which may lead the way to pathogen-specific vaccine development [150]. Adjunctive IFNA treatment in chronic HIV patients resulted in modest reductions of viral load, but poor recovery of CD4+T cells, prompting the majority of IFNA clinical trials to stop for futility [151].

Anti-TNF agents such as antibodies, chloroquine, pentoxifylline, or thalidomide can be useful if administered with ART to prevent or treat certain forms of IRIS [152–154]. The use of prednisone has successfully utilized for the treatment of TB-IRIS to reduce acute symptoms in the short term [152,155]. However, a combination of corticosteroids with intermittent regimens of IL2 increased T lymphocyte deaths [156,157]. Immunotherapy with IL2 alone was insufficient to improve immune restoration in AIDS patients, thus, proved to be low efficacy [158]. A deeper understanding of the pathophysiology of immune reconstitution and the immunopathogenesis of IRIS would perhaps shed light not only on the choice of immunomodulators [159] but also on the timing of ART initiation [160,161].

## Kinetics of Immune Reconstitution in Allo-HSCT Recipients

Slow and dysbalanced immune reconstitution is a deprecatory issue for patients who undergo allo-HSCT, as it is associated with the increased risk of infection-associated mortality [162–170]. Allo-HSCT from HLA-matched sibling donors (MSD) generally provides the best clinical outcomes and thus is regarded as the gold standard for transplantation [171]. However, because only one-third of patients have an MSD, many patients receive allo-HSCT from banked umbilical cord blood (UCB) [172]. In adults, the UCB transplantation is associated with lower rates of GVHD [172], but with a significantly higher frequency of viral infections and delayed immune cell reconstitution. UCB immune cells are considered more immature and antigen-inexperienced, which may explain the poor recovery of T cell immunity and the higher risk of viral infections caused by human Epstein-Barr virus, adenovirus, baculovirus, herpes viruses or cytomegalovirus (CMV) [173–176]. To evaluate quantitative immune recoveries several studies have been conducted [167,177–181].

Recent comparisons of immune reconstitution rates in 157 adult recipients who received MSD or UCB revealed that natural killer (NK) cells and B cells exhibited higher quantitative rates of recovery in UCB recipients during the first 6 months to 1 year after transplantation [178]. However, UCB recipients had slower T cell subset recovery, with lower numbers of CD3+CD8+ (naïve and effector), CD4+ (naive and memory), and regulatory T cells than MSD recipients from day 60 to one year of observation. Delayed quantitative recoveries of T cell most likely explain the increased rate of reactivation of latent viral infections in UCB recipients. The observation of rapid quantitative recovery of NK and B cells in UCB patients support the hypothesis that other immune cells synergize their effort to control latent infections, but in the absence of thymic function and the full recovery of T cells, the immune reconstitution is inefficient.

It is hypothesized that the increased rate of viral infections could be due to delayed quantitative or functional recovery of immune cells, or both. Cytomegalovirus (CMV) specific response can be used to evaluate the functional role of T cells derived from the homeostatic proliferation of the graft and the T cells generated by thymic T cell neogenesis in the adult recipient.

Several studies used enzyme-linked immune absorbent spot assay (ELISPOT) to evaluate the IFNG production by CD4+ and CD8+T cells toward CMV antigens [182,183]. Results showed that patients with high IFNG production were protected from developing CMV infection, whereas patients with low IFNG production were significantly more prone to CMV disease progression and in need of antiviral therapy. A study [90] showed that although naive cord blood T cells transferred to adult UCB recipients could initiate immune responses to CMV and become CMV specific effectors as early as 8 weeks after transplantation. Yet, they failed to clear CMV viremia. Thus, assessment of IFNG responses may be clinically relevant to differentiate the uneventful immune recovery from the pathological immune reconstitution that leads to viral reactivation.

## Treatment Approaches for Improvement of Allo-HSCT Immune Recovery

A number of immunotherapeutic approaches using T cell transfer to combat viral reactivation, improve the rates of immune reconstitution, as well as to prevent GVHD or disease relapse after allo-HSCT. There is clear evidence that infections can be treated by the adoptive transfer of T cells specifically targeting viral antigens [184]. Heslop et al. [185] showed that adoptively transferred EBV-specific cytotoxic CD4+ and CD8+ T lymphocytes can reconstitute the patient’s immune responses against EBV. Similarly, donor-derived adenovirus-specific T cells have been used for the treatment of patients with adenovirus infections after allo-HSCT [186]. Also, drug-refractory CMV infections after allo-HSCT have been successfully treated with CMV-specific T cells [187,188]. Invasive fungal infections, in particular, aspergillosis, represent another opportunistic life-threatening infection during immune reconstitution [189]. A clinical trial using aspergillus-specific T cell therapy showed suppression of antigenemia and prevention of invasive aspergillosis in a considerable number of patients [190]. Thus, the adoptive cellular immune therapy demonstrates high efficacy in restoring the anti-infectious T cell immunity after allo-HSCT [191].

Considering the importance of cytokine receptors in IFNG signaling cascade, several early phase clinical studies test cytokine agonist-receptor complexes. For example, IL15/IL15R complexes enhance immune activation in patients who relapsed within 60 days after allo-HSCT. The agonist complex was well-tolerated and did not increase the rate of adverse events [192]. Preclinical and clinical studies demonstrated the effectiveness of IL15 analogs to stimulate cytotoxic functions of CD8+ T cells and NK cells toward tumor antigens [193]. However, the limitation to its use is due to NK cell-mediated hyper-cytotoxicity though CD95, granzymes, and perforins, which is driven by abnormal IFNG production as shown in the settings of contact-dependent cardiac allograft rejection [194]. A phase 1 clinical trial of recombinant human IL7 (hIL7) in recipients of T cell-depleted allogeneic HSCT showed that CD3+, CD4+ and CD8+ counts are increased in hIL7 treated patients [195]. Exogenous administration of IL7 was found to enhance antigen-specific T cell responses to viral infections [196]. Thus, carries a promising potential for new treatment approaches for immune reconstitution disorders.

The efficacy of mesenchymal stem cells (MSC) transfer had been assessed to combat GVHD in ongoing clinical trials [197,198]. MSC are spindle-shaped multipotent progenitor cells with immunomodulatory capacities that reside in the bone marrow [199]. A pre-clinical study in mice showed that IFNG was required to initiate MSC efficacy. Recipients of T cells with poor IFNG secretion did not respond to MSC treatment and succumbed to GVHD. MSC, pre-treated with IFNG, became immediately active and suppress GVHD more efficiently [200]. Therefore, MSC may require activating signals from proliferating T cells to induce their suppressive effects. Gao et al. showed that repeated infusions of MSC might inhibit chronic GVHD symptoms in allo-HSCT patients, due to improved quantitative and functional recovery of T, B, and NK cell subtypes, leading to the acquisition of immune tolerance [198].

Adoptive transfer of Tregs has been additionally shown to be effective in the prevention and treatment of GVHD in preclinical models [201,202]. Tregs are able to inhibit immune responses without proinflammatory side effects and regulate immune cells from the adaptive and innate compartment including NK cells and antigen presenting cells (APCs) to prevent inflammation [203]. Clinical studies already revealed the potential of treatment with in vitro expanded Tregs [204–206]. Phase one clinical study presented that after Tregs transfusion, two of five patients showed clinical response with an improvement of chronic GVHD symptoms [205].

## Conclusion and Future Prospective

Deciphering the pathogenesis of immune reconstitution disorders remains a challenge [1]. Lower levels of interferons, but higher levels of other cytokines have been suggested as a risk factor. Thus, it becomes rational to consider the possibility of simultaneous supplementation of some cytokines and neutralization of the others to provide long-term control of inflammation [207].

Adjunctive IFNG treatment has been investigated with various outcomes, depending upon regimens. Interferons are potent inducers of other cytokines and numerous interferon-response genes, many of which are key hematopoietic transcription factors. However, the chronic and excessive production of IFNG or repetitive supplementation with downstream cytokines (e.g. IL15) induces cell exhaustion, bone marrow failure, accompanied by anemia [208]. The definitive data supporting the beneficial effect of interferons in host protective immunity during homeostatic repopulation are still lacking.

There still several unanswered questions concerning the role of interferon networks during immune recovery [209]. Which phase of immune reconstitution would benefit from interferon therapy the most? Which cell type should be targeted early during immune reconstitution, and which one is later? Are systemic interferon biomarkers as informative as those measured at the site of inflammation? In cells, the secretion of endogenous interferons and expression of interferon-response genes can be regulated by other molecules through transcriptional, posttranscriptional, and posttranslational mechanisms [132,210–214]. Thus, the investigations to search for novel drugs to timely alter the kinetics of immune reconstitution will have significant implications to optimize outcomes [215].

The evidence suggests that immunoregulatory success will depend not only on suppression of inflammation but also on re-directing the immune response toward resolution. The assessment of metabolic signaling in the maintenance of immune homeostasis and the settings of immune reconstitution is also under intense investigation [216]. Assessment of host IFN or IFNR genes polymorphism as factors that influence thymic recovery and resistance to latent infections may lead to the development of novel therapeutic strategies to combat immune reconstitution disorders [217].
